# Right Here Right Now (RHRN) pilot study: testing a method of near-real-time data collection on the social determinants of health

**DOI:** 10.1332/174426417X14987303892451

**Published:** 2018-05-31

**Authors:** Lynn Naven, Greig Inglis, Rachel Harris, Gillian Fergie, Gemma Teal, Rebecca Phipps, Sally Stewart, Lorna Kelly, Shona Hilton, Madeline Smith, Gerry McCartney, David Walsh, Matthew Tolan, James Egan

**Affiliations:** Glasgow Centre for Population Health, UK; University of Edinburgh, UK; NHS Greater Glasgow and Clyde, UK; University of Glasgow, UK; Glasgow School of Art, UK; University of Glasgow, UK; Glasgow Caledonian University, UK; NHS Greater Glasgow and Clyde, UK; University of Glasgow, UK; Glasgow School of Art, UK; NHS Health Scotland, UK; Glasgow Centre for Population Health, UK; University of Glasgow, UK; Glasgow Centre for Population Health, UK

**Keywords:** policy, evidence, real-time, technology

## Abstract

**Background:**

Informing policy and practice with up-to-date evidence on the social determinants of health is an ongoing challenge. One limitation of traditional approaches is the time-lag between identification of a policy or practice need and availability of results. The Right Here Right Now (RHRN) study piloted a near-real-time data-collection process to investigate whether this gap could be bridged.

**Methods:**

A website was developed to facilitate the issue of questions, data capture and presentation of findings. Respondents were recruited using two distinct methods – a clustered random probability sample, and a quota sample from street stalls. Weekly four-part questions were issued by email, Short Messaging Service (SMS or text) or post. Quantitative data were descriptively summarised, qualitative data thematically analysed, and a summary report circulated two weeks after each question was issued. The pilot spanned 26 weeks.

**Results:**

It proved possible to recruit and retain a panel of respondents providing quantitative and qualitative data on a range of issues. The samples were subject to similar recruitment and response biases as more traditional data-collection approaches. Participants valued the potential to influence change, and stakeholders were enthusiastic about the findings generated, despite reservations about the lack of sample representativeness. Stakeholders acknowledged that decision-making processes are not flexible enough to respond to weekly evidence.

**Conclusion:**

RHRN produced a process for collecting near-real-time data for policy-relevant topics, although obtaining and maintaining representative samples was problematic. Adaptations were identified to inform a more sustainable model of near-real-time data collection and dissemination in the future.

## Background

In order to improve population health and reduce health inequalities, it is important to understand the impacts of the social determinants of health, which span all areas of public policy and are well described (Dahlgren and Whitehead, 1993). The social determinants of health include unequal distributions of wealth and power, housing and the built environment, employment and working conditions, investment in the early years of life and social protection systems (Marmot et al, 2008). Researchers can support policymakers to address health inequalities by providing data and evidence on these social determinants of health.

However, despite increasing acceptance of the importance of evidence in influencing or determining policy, the dynamic relationship between policymakers and researchers continues to frustrate both groups in equal measure (MacGregor, 2013; Macintyre, 2012; Sanderson, 2011; Whitehead et al, 2004). These tensions arise across a range of dimensions including the dated nature of much of the available data, the use and misuse of particular research designs and types of data for different research questions, and the competing priorities of different groups involved in translating research into action (Smith, 2007; Petticrew et al, 2004).

Improving our understanding of how best to translate research findings into practice is an important research area (Macintyre, 2012; Barr et al, 2015; Smith and Katikireddi, 2013). It has been suggested that policymakers have described five forms of evidence to be particularly persuasive. These are: observational evidence showing the existence of a problem; narrative accounts of the impacts of policies from the household perspective; controlled evaluations; natural policy experiments; and historical evidence (Whitehead et al, 2004). A further study has suggested that research is most helpful to policymakers when it saves policymakers’ time; it is coherent and flexible enough to meet the particular policy needs at that time; the authors are regarded as credible; and the findings are succinct, clear and numerical (Stewart and Smith, 2015). The value of ‘bringing the data alive’ by providing real illustrations and ‘stories’ has also been described as useful. These findings suggest some of the complexities around policymakers’ use of evidence, where different stakeholders have different evidence needs and prioritise different forms of evidence which can be potentially challenging for gathering evidence (Brownson, 2009).

These challenges for researchers have coincided with increased opportunities created by the rapid rise in the use of portable electronic devices. Advances in technology have created a growing number of options for capturing context-specific, near-real-time data on people’s thoughts, behaviours and everyday experiences (Murthy, 2008; Martin et al, 2013). There is therefore potential to address some of the shortcomings of traditional research approaches (particularly in relation to timeliness, flexibility, convenience for respondents, and context appropriateness, as well as reducing recall bias) using technological innovation (Jones et al, 2012; Stone et al, 2007; Jones and Johnston, 2011; Kuntsche and Labhart, 2013). It is argued that repeated collection of real-time data may be particularly useful in illuminating the frequent, routine and mundane ‘lived experiences’ that are often hard to capture accurately through retrospective interviews, but which are crucial to understanding how people actually experience events (Jones et al, 2012). While online technologies have been identified as an important means of facilitating civic engagement (Coleman and Gotze, 2001; Oxman et al, 2009), there remains a gap in developing a definitive model for ongoing dialogue between citizens, researchers and policymakers using these technologies to maximise insights to inform current health policy debates.

For example, there is currently a lack of evidence on how near-real-time data might be collected and analysed to inform policy, and uncertainties about how new technologies could be more widely used to capture people’s everyday experiences. These uncertainties exist at several points in the process of gathering timely evidence to make the case for policy decisions. Identifying a sample frame and collecting data without selection bias may be made more difficult using a repeated data-collection approach (Jones and Johnston, 2011). The burden on participants may be greater, and the risks of attrition and response bias accentuated, if the requests for information become too frequent or unwelcome (Jones and Johnston, 2011). The balance between the speed of processing and analysing data, and the production of robust and accurate findings, is also uncertain. Finally, the capacity for policymakers to identify research questions amenable to this approach, and to use the outputs meaningfully, is untested.

The Right Here Right Now (RHRN) study was established to pilot a method of capturing people’s ‘lived experiences’ within a rapidly changing social and economic environment that has seen austerity, welfare reforms, a more precarious labour market, rising household costs and constrained public spending (Taylor-Gooby and Stoker, 2011). There was particular concern that the rapidly changing social security system, including changes in the eligibility, conditions, penalties and value of different entitlements, would have harmful health consequences, and that these were not being identified quickly enough for policymakers to respond – particularly in Scotland where some powers are available to mitigate such changes (McCartney et al, 2013). Informing policymakers with near-real time data is one approach to identifying emerging issues. We also wanted to know how people experience and react to the introduction of new policies, and the realities of people’s work, family and community life.

RHRN set out to explore whether we could build on some of the traditional strengths of repeated-measures panel surveys, but augmented with the additional value of real-time data capture, and the use of technology (Jones et al, 2012; Stone et al, 2007), in order to provide timely evidence to policymakers on the impacts of the socio-economic drivers of health and wellbeing. RHRN was a multi-centre collaboration between the Glasgow Centre for Population Health, NHS Health Scotland, the MRC/CSO Social and Public Health Sciences Unit, the University of Glasgow and the Institute of Design Innovation at the Glasgow School of Art.

This paper reports on the development of a methodology for gathering near-real-time data, and the conduct and outcomes of a six-month pilot study, which was designed to test several different functions in parallel, including:

- methods of sampling, recruiting and retaining study participants
- methods for question generation, data collection, analysis and dissemination of findings
- the ability to provide insights into participants’ ‘lived experiences’ and perceptions of topical policies and events
- the ability to provide data to stakeholders that have value and utility to inform decision making

## Methods

### Scoping and designing

The first stage of the project involved hosting a series of workshops with a focus on defining the scope of the project and informing the design of a potential pilot study. Ethical approval for this scoping and design phase was obtained through the Glasgow School of Art Research Office (reference number ET14001), and invitations were issued through a broad range of partner networks, outlining the main areas of interest of the study and aims of the workshops. The first workshop was undertaken with 50 invited stakeholders representing policymakers working in areas relevant to the social determinants of health, including the Scottish Government, local government, voluntary organisations, health boards and public health practitioners.The purpose of the workshop was to clarify whether there were unmet research needs that could be met by near-real-time data, and to explore ways in which these needs could be met. These stakeholders, while conscious of the time-lags in data collection, recognised that there needs to be a balance between near-real-time methods and depth of data, and generally favoured the collection of data that could furnish insights into ‘lived experiences’ and allow live issues to emerge. They also noted the importance of capturing the individual contexts of respondents to facilitate data interpretation, while at the same time expressing the need to recruit samples representative of the populations of interest.

To represent potential participant perspectives on the study, a further seven workshops were held in public locations and diverse communities in Glasgow, involving a total of 150 members of the public ranging in ages between 18 and 75 years and from a broad spectrum of socio-economic backgrounds. Settings included community halls, a street gala and a homelessness project. These were used to ascertain how best to engage and retain a panel of participants and disseminate results back to the panel. Overall, these participants favoured an approach that would allow data to be imparted quickly ‘on the go’ via mobile devices, which precluded gathering in-depth experiential data. In addition to their interest in a broad range of digital and social media platforms, they also highlighted the need for using more traditional approaches, such as telephone, email, text message, post and conversations facilitated by citizen researchers, to maximise inclusivity. There was also an interest in receiving instant visually attractive feedback based on their responses, including feedback from policymakers.

Some additional features proposed by the project team (such as carrying out nested studies with subsamples of participants to explore emerging issues of interest in more depth; extensive use of photographs; and targeting some questions to specific groups) were not possible because of time constraints during development and implementation and subsequently, for ethical and sample size reasons.

Decisions on study design were based on compromises between the requirements of these stakeholders and proxy participants. The resultant design comprised four-part questions delivered by: Short Message Service (SMS or ‘text message’); postal delivery of paper versions; and via a bespoke website. The paper version was designed in response to feedback at the workshops with members of the public regarding the need to address digital exclusion and ensure inclusiveness. These prototypes, alongside examples of a ‘findings summary’, information sheets and recruitment documentation were then all tested at two further community workshops and further refined, based on feedback.

Following the scoping and design phase, ethical approval was granted for a pilot study by the College of Social Science Research Ethics Committee at the University of Glasgow (application number 400140077). The pilot study was carried out between May and October 2015.

### Sampling, recruitment and retention

We piloted two parallel sampling strategies with a target sample size of 100 in each arm, which was deemed sufficient to address the needs of a small pilot study. First, a random clustered probability sample was identified from the postal address file for the city of Glasgow. This was chosen to test whether we could recruit a sample of people that would be representative of the population of Glasgow and thereby avoid biases associated with other sampling techniques (Bryman, 2008). The addresses for sampling were identified by stratification into deprivation deciles (using data for Glasgow taken from the Scottish Index for Multiple Deprivation (SIMD)) (Scottish Government, 2012), followed by random selection of sampling points (census output areas) within them. Based on an expected 33% recruitment rate, a total of 300 addresses were selected with a further top-up sample of 100 addresses drawn and held in reserve.

Each household received a letter introducing the study and was provided with a freepost return envelope for recipients to opt out of participating. Addresses that did not opt out were then visited by a fieldworker. Within each household, the potential participant was identified using the ‘last birthday’ method (Lavrakas, 2008). A protocol was developed for visiting households based on established procedures for survey research (Scottish Household Survey, 2015). Up to five attempts were made to establish contact at each address (at different times of day and including one day at the weekend). If there was no answer on the first visit a card with contact details was delivered. Once the individual within the household who had the last birthday had been identified, a maximum of three call-backs were made to each property to attempt to recruit that individual.Training was provided to all fieldworkers to ensure a professional approach to householders, sensitivity to any concerns they may have, and care in securing informed consent. Random sample recruitment commenced on 27th April, 2015 and was concluded on 19th July.

Second, a quota sample (based on the age, gender, ethnicity and area deprivation distribution of the city) was recruited from seven ‘pop-up’ stalls in diverse public locations across the city. The purpose of the quota sample was to test how design and engagement methods could be used to recruit a diverse group of participants. Individuals were eligible if they were aged >18 years, could read and speak English and were able to consent. For the probability sample, individuals had to be ‘usually resident’ at the sampled address, while in the quota sample individuals had to reside in Glasgow. Quota sample recruitment commenced on 26th April and concluded on 21st May.

At the point of recruitment, written consent, contact details and baseline socio-demographic information were recorded by fieldworkers. Prospective participants were given a unique user ID and password to the RHRN online system and guaranteed anonymity.

### Retention of study participants

Participants who did not respond to three weeks of consecutive questions were telephoned to ask if they wished to continue in the study, and to investigate any barriers to participation, so that they could be offered a change in the method of receiving and responding to questions. If no contact was established, and if non-response continued, up to two further attempts to call the participant were made. If participants did not respond for eight consecutive weeks and had not contacted the research staff, they were removed from the sample. After week 12, when we hosted our final pop-up recruitment event, we stopped removing participants for non-response to maximise the total possible respondents.

### Question process

The overall process included question generation, data collection, data analysis and dissemination of findings.

### Question generation

There were three sources of weekly questions:

- The stakeholders involved in the initial design process were emailed each week to ask if they had any questions amenable to this approach, based on their current priorities.
- Questions were generated by the research team where there was a relevant topical news item, or to coincide with particular calendar events.
- Questions were drawn from a pre-developed question ‘bank’ drawn up by the research team.

Where no stakeholder question suggestions were received, and where there were no topical news stories to draw on, questions from the ‘bank’ were chosen in an order that would ensure variability and maintain engagement of participants. After the initial topic area and general question was identified each week, a sub-group of the research team wrote initial questions and piloted these with other members of the research team and then with a small panel of ‘testers’ within the collaborating institutions to ensure that the questions flowed and were being interpreted as intended. As all questions were short, and delivered in four parts, they were not subjected to a readability assessment. The full list of questions is available in Appendix 15 of the RHRN report and, for an abbreviated list, see Table 2 (Fergie et al, 2016a).

### Data collection

**Figure 1:**
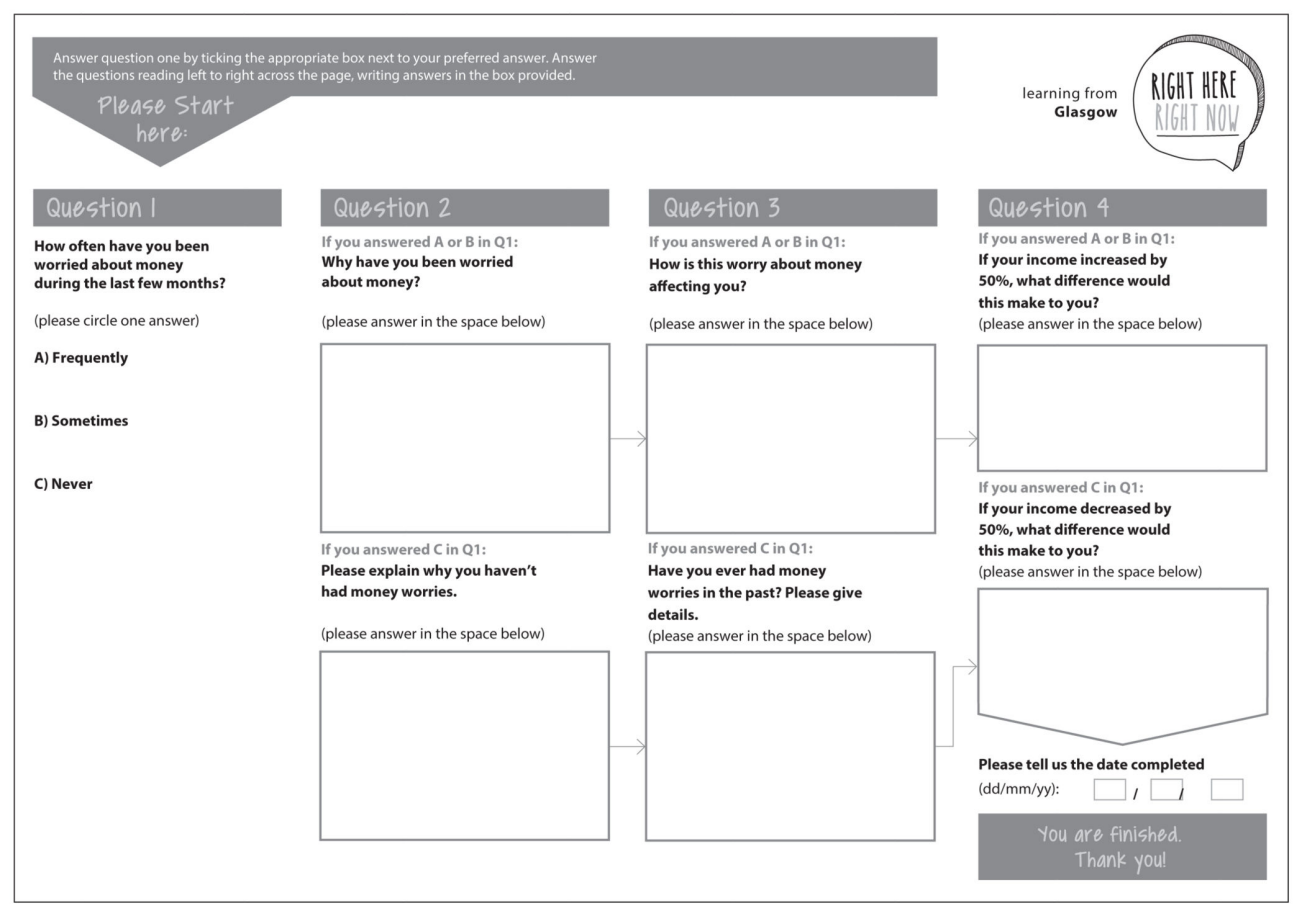
Example of a question on money worries issued during the RHRN pilot study

Participants were offered a choice of three methods of receiving questions: SMS (text messaging), email and post. Email respondents and smartphone users received a link to the bespoke website to enable them to answer directly online. Basic mobile phone users could reply using a free-to-end-user messaging service, and postal respondents received a paper questionnaire with a reply-paid envelope. Questions were issued weekly to participants and followed a four-part format, designed to facilitate increasingly deeper exploration of topics to generate more in-depth data. Question 1 was a multiple choice question to help tailor the follow-up questions; questions 2 and 3 asked for more detail about the response to question 1; and question 4 was designed to be open and creative and in some cases offered participants the opportunity to upload relevant photographs. See Figure 1 for an example question in postal questionnaire format, which was A3 size folded for posting. The reverse side contained an introduction, and information and contact details about support organisations.

Those who uploaded photographs were contacted for consent for the research team to use them. A total of 26 weeks of data were collected.

### Data analysis and dissemination

The bespoke website was designed to automatically generate high-level descriptive statistics of responses to the multiple choice quantitative questions, including response rates and a breakdown of the responses to each quantitative question. Thematic analyses of the qualitative data were carried out by the research team and findings summaries comprising the quantitative findings, key themes and illustrative examples identified from the qualitative data were produced each week, for dissemination to participants and stakeholders two weeks after the initial question was issued.

### Evaluation

An evaluation was undertaken to examine the methods used for all the pilot processes. This comprised an analysis of the representativeness of the achieved samples, recruitment and retention, weekly response rates and the question process.

To evaluate impacts, two questions were included as part of the weekly questions to participants, telephone interviews were carried out with a sub-sample of participants to gain insights into their experiences of the project, and a workshop with key stakeholders was held to capture their perspectives on the value and utility of the findings. The methods for the pilot study are more fully described in the published study report (Fergie et al, 2016a).

## Results

### Sampling, recruitment and retention

**Figure 2:**
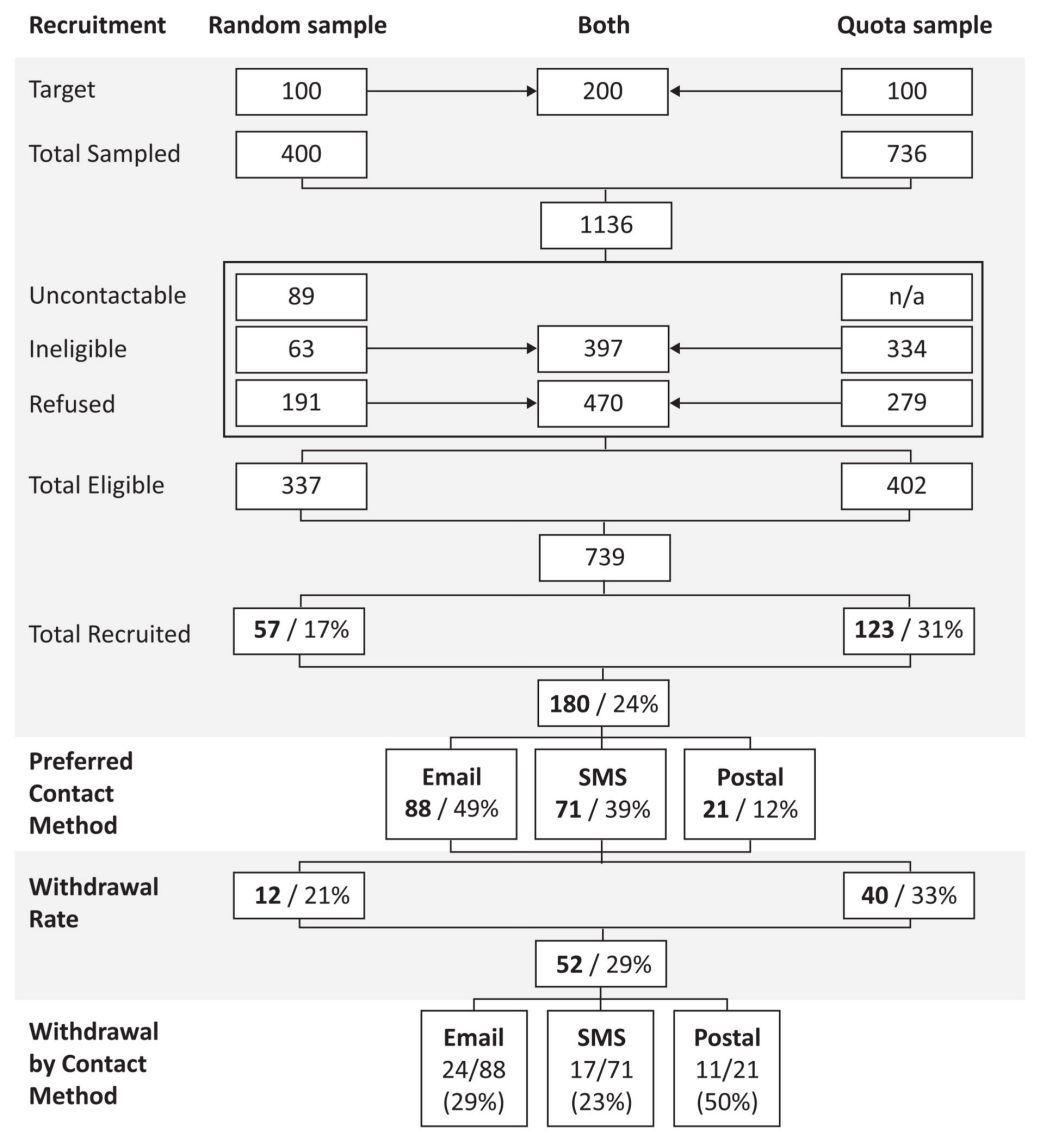
Sampling, recruitement and retention outcomes

Of the 400 addresses in the random probability sample frame, a total of 57 participants were recruited (17% of the eligible sample). In the quota sample, 736 people were approached, of which 402 were eligible and 123 were recruited (31%). Full details of the outcomes of sampling, recruitment and retention are outlined in Figure 2.

The recruitment of participants through the random sample was more labour-intensive than in the quota sample (7.2 versus 2.0 researcher hours per participant), and within the constraints of the small sample size, it could not be considered representative of the wider population. A substantially larger sample was achieved more quickly in the quota sample group. In terms of similarities with the Glasgow population, a summary of key demographic statistics from the random and quota samples compared with the Glasgow population is provided in Table 1.

Both samples were similar to the Glasgow population in terms of age and gender, but the random sample included fewer women and ethnic minorities than expected (although the differences to the Glasgow population were too imprecise to be certain). The quota sample was slightly more comparable with the Glasgow population on the characteristics that were included in the sampling frame (for example, age, gender, ethnicity and area deprivation) but showed bias on those aspects that were not included in the sampling frame. For example, both the random and quota samples under-represented individuals who did not possess any educational qualifications. More detail on the representativeness of the sample is provided in the RHRN report (Fergie et al, 2016a).

Over the course of the pilot, 12 (21%) and 40 (33%) people were withdrawn from the random and quota samples respectively. Of these 52 withdrawals from the study in total, eight contacted the research team directly and asked to be removed, and 17 asked to be removed as a result of a retention telephone call following a period of non-response. A further 27 participants were removed between weeks 8 and 12 of the study, following eight consecutive weeks of non-response, in accordance with the retention strategy. At withdrawal, participants were offered the opportunity to provide feedback on why they did not want to continue to participate in the study. Among the few who took this opportunity, most suggested they did not have time to contribute to the study on a weekly basis. When the final question was issued in week 26, 128 participants remained in the combined sample.

### Question generation, data collection, analysis and dissemination

#### Question selection

Over the 26 weeks of the pilot, nine weeks were used to ask questions identified by stakeholders, ten weeks for questions from the pre-determined question bank, which included two questions devoted to evaluation feedback, and seven weeks for questions in response to topical issues. Table 2 shows the breakdown of question topics and sources.

While the majority of questions related to the broad social determinants of health, as outlined in the Dahlgren and Whitehead model (1993; 2007), some others had a more tenuous link, such as a question on ‘Blood donation’, in response to National Blood Donation Week.

Topics such as walking, smoking in cars and travel around Glasgow linked to‘Individual lifestyle factors’; people, community, family and views on the refugee crisis linked to ‘Social and community networks’; access to public services, the quality of work, and heating the home related to ‘Living and working conditions’; and money worries, the Budget 2015, and credit and finance were connected to the ‘General socio-economic, cultural and environmental conditions’ that prevail in society.

#### Data collection

The most popular mode of participation was email (n=88, 49%), followed by SMS (n=71, 39%) and post (n=21, 12%). SMS respondents were less likely than email or postal respondents to be removed from the study (23%, 29% and 50% respectively) but SMS respondents were more likely to fail to respond to any questions than postal or email respondents (76%, 73% and 50% respectively).

The mean character count of responses to the qualitative questions was slightly lower for the SMS respondents than for the email or postal respondents (73, 98 and 103 characters respectively). The option of submitting photographs was available for email respondents for seven of the questions, and a total of six photographs were received. Overall, participants using email were the most likely to provide data over the course of the study. Although the pilot ran for 26 weeks, delays to recruitment and attrition meant that participants, from the random and quota samples, spent a mean of 19 and 18 weeks, respectively, in the study.

Over the course of the pilot, the mean response rate for the first question each week was 54% (range 47% to 64%) and 50% for the last question (range 41% to 58%). In total, participants at least partially responded to 45% of the weekly question sets issued to them.This figure was slightly higher in the random sample (51%) than the quota sample (42%), although this difference was not statistically significant.

#### Analysis and dissemination of findings

Due to the short turnaround time for thematic analysis of the qualitative data, results were presented in the form of brief summaries of findings, which are published in a project booklet (Right Here Right Now, 2015).

### Insights into participants’ perceptions

Questions generally concerned people’s perceptions of a range of topical policies and events and their ‘lived experiences’ of social and economic changes that could impact on health. Given that austerity, welfare reform and the changing labour market were the underlying contexts of the study, we were interested in some of the themes emerging from questions related specifically to these themes. This included questions on the (Chancellor of the Exchequer’s) 2015 budget, the quality of work, money worries, stress, credit and finance, and public services. An example of qualitative responses to the question on money worries is shown in Box 1.

The responses to the money worries question demonstrate the potential for the RHRN model to generate qualitative insights relevant to contemporary policy decisions, such as austerity measures.

### Insights into participants’ experiences of RHRN

Additionally, we were interested in participants’ experiences of taking part in RHRN, in terms of satisfaction with the format and frequency of questions, the topic areas, methods of engaging and whether the project engendered a feeling of being part of something important. Data from one of the two evaluation questions on the perceived importance of the issues we were asking about revealed that, of the 68 respondents to this question, the majority thought the topics were either ‘very important’ (47%) or ‘quite important’ (44%), while 9% considered them ‘not very important’. Many respondents felt they covered important areas of life that affect them. When prompted for views on how the RHRN questions should be decided, many participants believed that decision makers should give more of a voice to community members:

Just by asking local folk what they think is important to them and how we can help shape the future.

In response to a question about the frequency of the questions being asked, of the 76 people who responded, 84% were satisfied with weekly questions, while 15% felt that weekly was too frequent. One percent thought the weekly format was not frequent enough. The majority of people who took part in follow-up participant interviews found the experience enjoyable and looked forward to receiving the questions. Several interviewees stated that they had varied interest in the question topics, but most reported that, regardless of the topic, they either felt a duty to answer, or they appreciated engaging with questions they would not otherwise have considered.

The diversity of the topics was quite interesting, maybe it made you stop and think a little bit about things – it just gave you a little prompt and maybe you would spend a little more time thinking about certain issues that maybe you wouldn’t normally think about.

Those who used the website, via email or smartphone, reported advantages in terms of convenience of being able to answer when it suited them, and scope to elaborate on their answers on the online template.

Participants who had accessed the findings summaries valued this rapid feedback on the questions which gave them the opportunity to compare their views with those of others. A consistent theme which emerged was that the study made participants feel engaged as citizens and provided them with a voice to speak to decision makers about current and important issues. This opportunity to be influential in shaping policy emerged strongly from the follow-up interviews and was cited as a key motivating factor in encouraging participation over a longer period of time. Some interviewees suggested that their motivation to continue participating over a longer period would be enhanced by feedback on how the data were being used by decision makers.

### Value and utility of data

Several themes emerged from the stakeholder evaluation workshop which reflected the value and utility of the findings to inform decision making.

In terms of the presentation of results, stakeholders viewed the findings summaries as visually appealing and accessible, and valued the insights they gave into perceptions of Glasgow residents on a range of current issues. From the point of view of utility, it was thought that the findings, in their current format, had potential to highlight and raise the profile of emerging issues that might not otherwise receive attention until evidence is gathered in more traditional ways. It was also suggested they could inform calls for evidence and national consultations, and contribute to local priorities.

An initial ambition was that we could carry out comparative analysis based on participant demographic data, and also integration of RHRN findings with existing datasets. While time constraints, and lack of comparability of RHRN questions with those used in some more traditional surveys and approaches, limited this during the pilot, the ‘refugee’ topic demonstrated the potential for comparisons with other data sources. On the question of whether we should welcome more refugees, the majority of RHRN participants (60%) felt we should, which was consistent with an Ipsos MORI poll published in September 2016 showing that 57% of Scottish people were ‘confident that most refugees who come to the UK will successfully integrate into their new society’ (Ipsos MORI, 2016). The main motivations cited by RHRN participants for welcoming more refugees were on moral and humanitarian grounds.

The percentage of RHRN participants who thought we should not welcome more refugees (21%) was also relatively compatible with the 27% in the national poll who were of the opinion that ‘we can’t accept any at this time’. This demonstrated the potential for a process like that of Right Here Right Now to respond to rapidly changing current events.

Although there was substantial interest among decision makers about the potential of near-real-time data, it was not yet clear to them how they could use such a resource effectively (either in terms of asking questions or using results). Some concerns were raised about the potential sampling and response biases in the samples, and the impact that would have on the validity of the quantitative results. The small sample size precluded analysis of results by demographic characteristics of participants, which was a stated preference of stakeholders during the workshop phase. However, it was acknowledged that RHRN was a pilot delivered over a short timescale and, as such, subject to some shortcomings. In the longer term, stakeholders felt that, to overcome the lack of representativeness of findings, RHRN would need to provide deeper qualitative insights into people’s experiences through more in-depth studies, although it was not clear how such data would have been used.

Stakeholders were asked if the immediacy of RHRN findings could fit within their practice, from the point of view of having the capacity to deal with ‘real-time’ evidence. Some alluded to difficulties around exploiting real-time data opportunities, as their decision-making processes are aligned to longer-term strategies and are therefore not flexible enough to respond to such rapid evidence generation. The general view was that a turnaround of 6–8 weeks would be sufficient to generate research information that was ‘timely’ but not necessarily ‘real-time’ *per se*.

Additionally, the independence of the data was valued by stakeholders and gave the findings greater credibility.

## Discussion

### Summary of main findings

The RHRN pilot study attempted to find a means of utilising technological developments to provide near-real-time quantitative and qualitative data to inform decision-making processes. A panel was recruited and retained over 26 weeks, but sampling biases meant results were not representative of the wider population, and thus produced insights of a more qualitative nature. Overall, however, the study generated data which were of substantial interest to stakeholders. The project was also valued by participants, in terms of giving them an opportunity to have their voices heard and a perceived sense of responsibility or status as a result of being consulted every week.

### Strengths and weaknesses

The major strength of the project was that it succeeded in generating a system for collecting near-real-time data on relevant topics and subsequently disseminating the findings. Questions were produced quickly in response to topical events and requests from stakeholders. It proved possible, if resource intensive, to invite, design, approve and upload questions to the website and paper template, and then to collect and analyse the data, and provide summaries to stakeholders and research participants – all within a two-week period. However, these weekly processes meant it was not possible to perform in-depth analysis of the qualitative data, resulting in summaries of findings that were general and brief in content. Additionally, despite having collected rich demographic data from participants at recruitment, comparative analysis of responses based on this background data was not possible, nor was it possible to situate the emerging findings within wider theoretical and empirical contexts. However, the study provided important new learning around the definition of ‘real-time’, suggesting that such a rapid turnaround of data was not deemed essential to fit with decision-making processes. Therefore, within a longer 6–8 week timeframe, there would be potential to compare the findings with existing evidence around the themes being explored.

Notwithstanding these limitations, stakeholders highly regarded the rapid findings summaries in terms of providing accessible, visually appealing results quickly. They also valued the insights the summaries provided into perceptions of Glasgow residents on a range of topical and current issues, and offered a number of suggestions as to how the findings could be utilised to add value.

The pilot relied heavily on the website developed for use with the participation methods to ensure consistency of questioning and automation of question issue, response data management, high level response rate statistics, and dissemination of findings. The use of SMS placed restrictions on the number of characters that could be sent in one message (without being split into multiple messages leading to confusion), and it was not possible to find a free-to-end-user photo messaging service with SMS, which meant that only email participants could choose to respond with photographs. For a larger pilot, more investment would be required to develop a more flexible system. Additionally, more time to develop the technological interface for participants may have increased the possibility of gathering a greater depth of qualitative data, and of greater variety (for example, audio data).

The pilot also illustrated the challenges of recruiting a representative sample to take part in a repeated measures panel study like RHRN. Findings from other online panel studies using random sampling demonstrate varied response rates, ranging from a low of 10% to a high of 48% (Hays et al, 2015), indicating the potential to learn from these studies if the aim of a future study is to recruit a sample that is representative of the wider population. The quota sample was selected on four population characteristics and was therefore unlikely to match the population on variables not included in the sample framework. While there is always inherent bias in quota sampling, the relative speed and efficiency of this sampling method suggests scope for targeting potential participants on the basis of their particular experiences related to questions of interest.

A significant limitation of the pilot study could be attributed to its ambitious nature in terms of addressing the multiple aims discussed, and the conflicting requirements expressed by diverse stakeholders and participants. This echoes similar observations about the value of different forms of evidence to different stakeholders (Brownson et al, 2009). The resultant compromises that had to be made, alongside the short timeframe for development, further impacted on the study methodology. Future studies exploring the value of rapid data collection and analysis could benefit from more focused aims and further preparatory work to understand how best to inform decision-making processes.

### Implications

Right Here Right Now was a co-produced study involving extensive engagement with a broad range of stakeholders and the public. It sought to meet the expressed needs of decision makers for more rapid research findings and exploit opportunities presented by technological developments. In these respects the study was very successful.

Having good stakeholder relationships was important to the development of the pilot and the generation of relevant topics for questions, and made it more likely that the findings could be used and linked to decision-making processes. This aspect of the study could potentially be strengthened if a longer lead-in time had been available, so that stakeholders could more fully realise the value of near-real-time data and integrate it into their decision-making processes.

If the demand for near-real-time data persists in the public health community, and if the required data are high-level indications of reactions to, or experiences of events, or data not yet collected by other means, then the current RHRN approach could fill this gap. A key finding of this research is the perceived value of near-real-time data, and further clarification of what is considered to be ‘real time’. Given the reports from stakeholders that their decision-making processes cannot respond to weekly evidence, there is scope to expand the definition of ‘real-time’ to allow for more in-depth data collection, analysis and reporting, which may align better with those processes. From the point of view of decision makers, the key benefit was evidence which did not have the lag time of more traditional surveys, or which fitted within particular process timescales, such as consultations. A very rapid turnaround was not deemed essential for this and ‘real time’ could therefore be considered as a 6–8 week window in terms of informing decision-making processes. Due to the limitations around the depth of data emerging as part of the study it may be that, if this is the preferred approach, nested qualitative approaches using more traditional designs may be required to achieve this. Within a 6–8 week timeframe, further exploration of ‘lived experiences’ through nested studies would be more realistic. Whichever approach is adopted, further studies would require a longer timeframe for development and delivery of the process, and larger sample sizes to ensure confidence in the findings.

From the point of view of participants, rapid feedback on the questions was valued as it provided reassurance that their views had been listened to, and a summary of feedback was made available within a timescale that made it feel dynamic and relevant. Additionally, it was suggested by some that being clearer about who was asking the question and, particularly, how the findings were being used by decision makers, would improve the experience and provide even greater motivation to participate. This suggested potential for this design to support citizen involvement in policy- and decision-making processes. Given that participants valued the opportunity to be given a voice and indicated an interest in helping to shape decision making, this study suggests potential to align with current Scottish Government policy, developed to ensure that public service delivery is shaped around the needs and demands of individuals and communities (Scottish Government, 2010). This public service reform is reinforced by the Community Empowerment Act (Scottish Government, 2015), which advocates co-production approaches to strengthen community voices in decisions that affect them.

This study highlighted how tensions arise in the development of a system which aims to be innovative in using online technologies to generate data rapidly, while maintaining an inclusive approach for individuals with limited access to such technologies, and adhering to ethical principles of data protection and anonymity. Many approaches to public participation in policymaking are deliberative in nature, such as citizens’ juries (Smith and Wales, 2000). These are most suited to generating insights from an informed sample of citizens on a contentious policy problem (Degeling et al, 2017), unlike RHRN which sought participants’ insights, in near-real-time and on an ongoing basis, in response to a range of contemporary events and rapidly changing social and economic circumstances. With further technological development, potential exists for the RHRN approach to be better tailored to align with existing and innovative methods of knowledge exchange where, instead of manually coordinating topic suggestions and requests from stakeholders, RHRN could draw on communities of practice as they develop around relevant knowledge-exchange portals (Quinn et al, 2014), to identify emergent evidence gaps.

### How it fits with existing literature

Real-time surveillance systems are used frequently to identify health protection issues (Chretien et al, 2009; Miaux et al, 2010), but to our knowledge this is the first attempt to create a near-real-time system for informing policy on the social determinants of health. The rapid growth and widespread adoption of new media have meant that developing such a system remains under-exploited as a tool for informing public policy and engaging the public with research (Househ, 2014). While research communities have yet to fully harness the potential of new media and near-real-time data collection as a means of connecting policymakers with the public, online communities are well established, particularly around key health issues. This includes provision of peer support for particular health conditions (Eysenbach et al, 2004; Fergie et al, 2016b), facilitation of health-related political activism (Labonte, 2013), and contributions to contemporary health debates (King et al, 2013; Dyar et al, 2014). The additional value of new media is thought to lie in its potential for engaging the public with research (Househ, 2014) and policymaking (Langston et al, 2005; Oliver et al, 2004), as a means of increasing the relevance, quality and transferability of research. Drivers for engagement also stem from a ‘social commitment’ to open up dialogue and democratise scientific and policymaking processes (Caron-Flinterman et al, 2005; Delgado et al, 2011).

Given the rapid changes in the communication landscape brought about by internet use and new media, this study has been overdue and important in developing a more nuanced understanding of the potential opportunities, challenges, and methodological issues related to creating a near-real-time system for exploring how people are responding to the current rapidly changing social and economic landscape.

## Conclusion

The RHRN pilot study demonstrated that it is possible to create a panel study in which questions can be developed and distributed, data collected and analysed, and results disseminated within two weeks.

The study demonstrated some good examples of the utility and value of such near-real-time data in contributing to consultations and calls for evidence, and in responding to stakeholder priorities.The evaluation identified a range of areas where adaptations could be made to ensure a more sustainable model of near-real-time data collection, interpretation and dissemination in the future, with greater potential to influence policy.These included more clearly defining the focus of the study, ensuring at the beginning that the methodology and process are aligned to these well-defined and compatible aims, and greater concentration on the development of a flexible technical platform to aid project delivery.

## Figures and Tables

**Table 1:** Comparison of RHRN participant demographics with Glasgow population

Socio-demographic characteristic	Categories	Random sample (n=57)	Quota sample (n=123)	Glasgow 2011 Census
Gender	Male	33 (58%)	56 (46%)	48%
	Female	24 (42%)	67 (54%)	52%

Age	18-29	16 (28%)	39 (32%)	27%
	30-44	17 (30%)	31 (25%)	27%
	45-64	18 (32%)	35 (28%)	29%
	65+	6 (11%)	18 (15%)	17%

Ethnicity	White	55 (96%)	110 (81%)	90%
	Non-white	2 (4%)	13 (11%)	10%

Educational qualifications	Degree level or equivalent	19 (33%)	49 (40%)	27%
	Mid-low level qualification	32 (56%)	61 (50%)	41%
	No qualifications	6 (11%)	10 (8%)	32%
	Missing	0	3 (2%)	0

Glasgow Index of Multiple Deprivation quintile	Most deprived	13 (23%)	26 (21%)	20%
	2	11 (19%)	28 (23%)	20%
	3	6 (11%)	20 (16%)	20%
	5	13 (23%)	18 (15%)	20%
	5 – Least deprived	13 (23%)	22 (18%)	20%
	Missing	1 (2%)	9 (7%)	0

**Table 2:** Question topics and sources

Stakeholder requests	Question ‘bank’ drawn up by project team	Topical/current news
People (population)	Heating	Walking
Community	Stress	Blood donation
Healthy ageing	Family	Budget 2015
Museums and art galleries	*Evaluation feedback* (views on project questions)	Quality of work
Commonwealth Games	Volunteering	Smoking in cars
Discrimination	Money worries	Refugee crisis
E-cigarettes	*Evaluation feedback* (experience of taking part)	Travel
Smoking ban	Public services	
Children (child-friendly city)	Credit and finance	
	Living in Glasgow	

## Box 1 Money worries question

The question on money worries revealed that 74% of the 80 respondents reported having been either ‘frequently’ or ‘sometimes’ worried about money in the previous few months. The main reasons given for this worry were insufficient money and rising costs: “not enough money to pay bills and for my son for his education, hobbies and haven’t had a holiday in over 7 years through not having money”

Some particular circumstances that led to money worries were debt and redundancy. For one participant who described constantly living on the breadline, this meant: “Having to work two jobs, constantly tired”

Others described effects on their emotional and mental health, including stress, sleeplessness, depression, loss of appetite and impacts on relationships: “I feel stressed and anxious. I have difficulty shutting off from thinking about money and my wife and I constantly bicker about money”

Wider effects on participants’ lives were also reported: “Every day in every way. Never enough to do the things I want, barely enough to cover what I need”; and “Feeling of not being in control”.
